# Prevalence and Impact of Partial Anomalous Pulmonary Venous Connection in Turner Syndrome

**DOI:** 10.1016/j.cjcpc.2025.06.004

**Published:** 2025-07-09

**Authors:** Estelle Tenisch, Silvia Gigliotti, Jenny Lam, Kanetee Busiah, Peter Kopp, Florence Niel-Bütschi, Nelly Pitteloud, Judith Bouchardy, Nicole Sekarski, Magalie Ladouceur, Tobias Rutz

**Affiliations:** a Pediatric Radiology Unit, Department of Medical Imaging and Interventional Radiology, Lausanne University Hospital and University of Lausanne, University of Lausanne, Lausanne, Switzerland; b Congenital Heart Disease Unit, Service of Cardiology, Lausanne University Hospital and University of Lausanne, Lausanne, Switzerland; c Paediatric Endocrinology, Diabetology and Obesity Unit, Women-Mothers-Children Department, Lausanne University Hospital and University of Lausanne, University of Lausanne, Lausanne, Switzerland; d Division of Endocrinology, Diabetes & Metabolism, Centre Hospitalier Universitaire Vaudois (CHUV), Lausanne, Switzerland; e Laboratory Medicine and Pathology Unit, Genetic Labs Department, Lausanne University Hospital and University of Lausanne, Lausanne, Switzerland; f Pediatric Cardiology Unit, Lausanne University Hospital and University of Lausanne, Lausanne, Switzerland

**Keywords:** Turner syndrome, partial anomalous pulmonary venous connection, cardiac MRI-bicuspid, aortic valve, congenital heart disease

## Abstract

**Background:**

Patients with Turner syndrome (TS) have a higher mortality than age-matched females, mainly due to cardiovascular disorders. Recently, an increased prevalence of partial anomalous pulmonary venous connection (PAPVC) was described in patients with TS. However, data on the clinical impact of PAPVC, that is, the need for surgical correction, are scarce. This study aimed at evaluating the prevalence and impact of congenital heart disease (CHD) with a focus on PAPVC in a TS population.

**Methods:**

Patients with TS of all ages were included. Clinical data and reports of echocardiography, cardiac magnetic resonance (CMR), or computed tomography (CT) were retrospectively evaluated. CMR and CT were reviewed for PAPVC.

**Results:**

Seventy-nine patients with TS were included (53 adults, 26 children [mean age 25 years, range 5-67 years]). Sixty-five patients underwent echocardiography, 41 CMR, and 3 CT. Cardiovascular disorders were present in 45% of patients and more frequently in karyotype monosomy X (76%), followed by 45, X mosaicism (39%), X structural rearrangements (18%), and other, unknown (16%) (*P* < 0.02). The encountered CHD lesions were bicuspid aortic valve (N = 16, 24%), PAPVC (N = 6, 8%), and aortic coarctation (N = 4, 6%). Nine (13.4%) patients underwent surgery, mainly for aortic coarctation repair (N = 4). Only 1 of the 6 patients with PAPVC needed surgery.

**Conclusions:**

The spectrum of cardiovascular disorders in TS appears to be associated with the karyotype. Among CHD lesions, bicuspid aortic valve is the most prevalent, followed by PAPVC, although the latter seems to have a minor impact on outcome. However, PAPVC is systematically missed on echocardiography and classically found in patients with complete monosomy X.

## Résumé

**Contexte:**

Le taux de mortalité est plus élevé chez les femmes atteintes du syndrome de Turner que chez des femmes appariées selon l’âge, principalement en raison des troubles cardiovasculaires. Des rapports récents font état d’une prévalence accrue de la connexion veineuse pulmonaire partielle anormale (CVPPA) chez les femmes atteintes du syndrome de Turner. Les données relatives aux conséquences cliniques de la CVPPA, à savoir la nécessité de recourir à une correction chirurgicale, sont néanmoins limitées. Cette étude visait à évaluer la prévalence et les conséquences des cardiopathies congénitales, et plus particulièrement de la CVPPA, dans une population atteinte du syndrome de Turner.

**Méthodologie:**

Des patientes de tous âges atteintes du syndrome de Turner ont été incluses. Les données cliniques ainsi que les résultats d’examens d’échocardiographie, d’imagerie par résonance magnétique (IRM) cardiaque ou de tomodensitométrie (TDM) ont été évalués rétrospectivement. Les résultats d’examens d’IRM cardiaque et de TDM ont été examinés pour rechercher la présence d’une CVPPA.

**Résultats:**

Soixante-dix-neuf patientes atteintes du syndrome de Turner ont été incluses (53 adultes et 26 enfants [âge moyen : 25 ans; plage : 5 à 67 ans]). Soixante-cinq patientes s’étaient prêtées à une échocardiographie, 41 à un examen d’IRM cardiaque, et 3, à une TDM. Des troubles cardiovasculaires étaient présents chez 45 % des patientes, plus fréquemment chez celles porteuses d’une monosomie X (76 %), suivies de celles porteuses de mosaïques 45, X (39 %) et de réarrangements structuraux du chromosome X (18 %), et pour un motif inconnu dans les cas restants (16 %) (*p* < 0,02). Les anomalies associées aux cardiopathies congénitales observées sont les suivantes : valve aortique bicuspide (n = 16; 24 %), CVPPA (n = 6; 8 %) et coarctation de l’aorte (n = 4; 6 %). Neuf patientes (13,4 %) ont subi une intervention chirurgicale, principalement pour corriger la coarctation de l’aorte (n = 4; 6 %). Seule une des six patientes présentant une CVPPA a nécessité une intervention chirurgicale.

**Conclusions:**

Le spectre des troubles cardiovasculaires observés chez les patientes atteintes du syndrome de Turner semble lié au caryotype. Parmi les anomalies associées aux cardiopathies congénitales, la valve aortique bicuspide est la plus fréquente, suivie de la CVPPA, même si les conséquences de cette dernière sur le pronostic semblent très limitées. La CVPPA échappe néanmoins systématiquement à la détection échocardiographique et est généralement présente chez les patientes présentant une monosomie X complète.

Turner syndrome (TS) is a sex chromosome disorder characterized by partial or complete loss of an X chromosome. It is the most common sex chromosome disease in women,^1^ with a prevalence of 1 in 2500 live births.^2^ Usually, TS can be identified during the prenatal period or in early childhood.^2^^,^^3^ The most common karyotypes include a complete X monosomy (45, X) or mosaicism.^4^

The key clinical features are short stature and hypogonadism, resulting in infertility. Dysmorphism and skeletal anomalies, autoimmune disorders, and renal complications are frequent comorbidities. Patients with TS have a mortality rate that is 3 times higher than that in age-matched females in the general population.^1^^,^^2^^,^^5^ Half of patients with TS present with congenital heart disease (CHD) and/or acquired cardiovascular diseases (ACVD).^4^, ^5^, ^6^, ^7^

The main ACVD in TS are aortic dilation and dissection, stroke, ischemic heart disease, hypertension, and arrhythmia, all occurring earlier than in the general population.^5^ ACVD has been recognized as the main cause of increased death in this population.^2^^,^^4^ Among all ACVD in TS, aortic dissection has the most important impact on mortality and morbidity.^1^^,^^8^ Moreover, women with TS have more cardiovascular risk factors than the general population, such as diabetes mellitus and hypercholesterolemia, which predispose to developing ACVD.^5^^,^^7^^,^^9^

Regarding CHD in TS, the most important entities include a bicuspid aortic valve (BAV), coarctation of the aorta (CoA), partial anomalous pulmonary venous connection (PAPVC), persistent left superior vena cava, and atrial and ventricular septal defects.^5^^,^^8^^,^^10^

PAPVC is characterized by the anomalous connection of some but not all pulmonary veins, to the right atrium and/or to the systemic vein. This left-to-right shunt increases blood volume in the right heart, which may cause right chamber dilatation and lead to right heart failure. Pulmonary arterial hypertension and arrhythmias are further possible consequences.^2^^,^^11^ One of 4 women with TS presents with PAPVC, and half of them develop right atrial and/or ventricular dilatation.^12^^,^^13^

However, information on the exact prevalence, precise anatomy, and clinical impact of PAPVC, in particular the need for surgical repair, remains scarce.^14^^,^^15^

Considering the high prevalence of CHD and ACVD, a cardiac examination (including electrocardiography, transthoracic echocardiography [TTE], computed tomography [CT], or cardiac magnetic resonance [CMR]) is recommended every 5-10 years to stratify cardiovascular risk in patients with TS.^9^

The aim of this study was to evaluate the prevalence of PAPVC and the associated karyotype. We also looked at the number of patients with PAPVC requiring surgery to correct this anomaly. The secondary aim of our study was to quantify the number of patients with TS with other cardiovascular anomalies requiring surgery, that is, BAV, aortic dilatation, aortic coarctation, and transposition of the great arteries (TGA), and to evaluate their karyotype. For all patients, we also determined right and left ventricular function and searched for valvular disease as secondary outcome.

## Methods

This is a retrospective observational cohort study.

The raw data that support the findings of this study are available from the corresponding author on reasonable request.

### Population

Patients with TS of all ages followed at our center (a tertiary center following pediatric and adult patients) were included in this study. A retrospective analysis of clinical data and reports of imaging studies acquired through the routine imaging protocol since 2000 when the PACS (picture archiving and communication system) was launched in our institution (TTE, CMR, and CT) was performed. In addition, CMR and CT images were reviewed for the presence of PAPVC. There is no systematic imaging follow-up for all patients with TS in our center, and it is dependent on the attending physician. This heterogeneity is also partially explained by the long inclusion period, during which practice patterns may have evolved.

Every patient with TS with at least 1 imaging examination was eligible, without exclusion criteria. All patients' medical records were available. Information such as ventricular volume and function, aortic size, cardiovascular malformation, valvular disease, and past cardiac surgeries was compiled after analyzing the medical record and reviewing all the available imaging studies. CT and magnetic resonance imaging (MRI) were reviewed by a pediatric radiologist with more than 15 years of experience. Echocardiography was reviewed by a cardiologist specialized in congenital cardiac malformation with more than 20 years of experience.

Seventy-nine patients were found in our database. Twelve were excluded because of absent imaging studies.

Sixty-seven patients with TS between 5 and 67 years old were included in this study. There were 48 adults (mean age: 32 years) with 48 TTE and 39 MRI. There were 19 children younger than 18 years (mean age: 11 years) with 1 MRI and 18 TTE.

The patient cohort was then divided into 4 groups according to their karyotype: (1) "complete monosomy X" (45, X), (2) "45, X mosaicism," (3) "X structural rearrangements," and (4) "other/unknown" for patients without known or very rare karyotypes. There were no patients with structural abnormality of the Y chromosome.

### Information and consent of participants

The study protocol was evaluated and approved by the local ethics committee. Need for written informed consent was waived for both pediatric and adult patients.

### Statistical analyses

Normal distribution of continuous variables was tested with the Kolmogorov-Smirnov test. Frequency analyses were used to describe the cohort and the prevalence of PAPVC and other cardiovascular lesions. To describe differences between groups of patients with and without PAPVC and groups of karyotypes, continuous variables were analyzed by the Student *t* test or by the Mann-Whitney test, where appropriate. Comparison of categorical variables was performed using the χ^2^ test or the Fisher exact test, where appropriate. Odds ratios (OR) and with 95% confidence intervals (95% CI) were calculated for evaluation of effect size. All tests were 2-tailed, and the statistical level was set to a *P* value of <0.05. Statistical analyses were performed using SPSS (version 27.0, Statistical Package for the Social Sciences; International Business Machines, Inc, Armonk, NY) and Excel (Microsoft, Redmond, WA).

## Results

### Population

The patient characteristics are summarized in Table 1. All but one child underwent TTE. All adults had a TTE. Among them, CT was performed in 6.3% (3 of 48) of the patients and MRI in 79.2% (38 of 48). Cardiovascular abnormalities were present in 40.3% (27 of 67) of patients. The most frequent CHD lesions were, in decreasing order of prevalence, BAV, aortic dilatation (defined as a diameter ≥2 scores for children or >20 mm/m^2^ for adult patients), PAPVC, and CoA. TGA was observed in 1 patient.

**Table 1:** Patient characteristics

Characteristic	Total (N = 67)	Surgical intervention
Cardiovascular disorders, N (%)	27 (40.3)	9
Bicuspid aortic valve, N (%)	16 (23.8)	3 (1 Ross procedure, 1 aortic dissection repair, 1 aortic root + aortic valve repair)
Aortic dilatation (>2 SD for children or >20 mm/m^2^ for adults), N (%)	13 (10.3)	
Partial anomalous pulmonary venous connection (PAPVC), N (%)	6 (9)	1 (PAPVC correction)
Aortic coarctation, N (%)	4 (6)	4 (coarctation repair)
Transposition of great arteries, N (%)	1 (1.5)	1 (switch)

SD, standard deviation.

### Influence of karyotype

**Figure 1:**
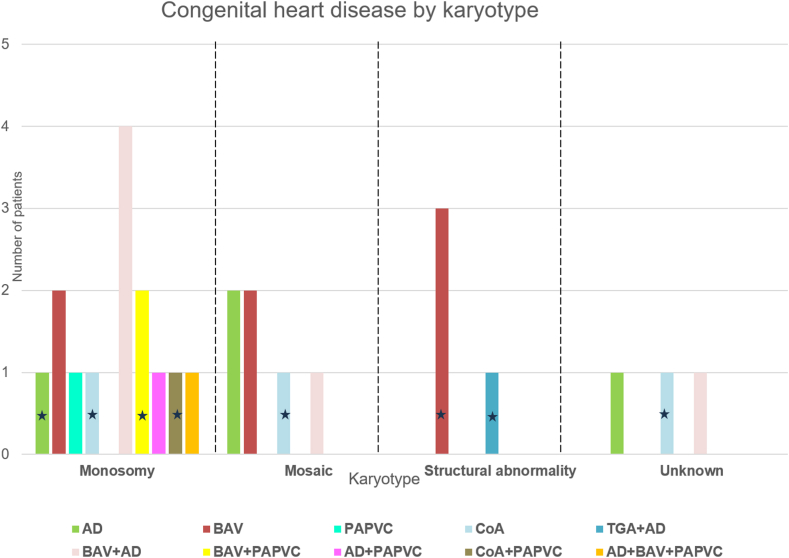
Repartition of karyotypes in the 27 patients with cardiac abnormalities. Twenty-seven of the 67 patients had a congenital heart disease. ∗Patients who required surgery. AD, aortic dilatation; BAV, bicuspid aortic valve; CoA, coarctation of the aorta; PAPVC, partial anomalous venous connection; TGA, transposition of the great arteries.

Twenty-seven of the 67 patients with available imaging had a diagnosis of CHD, distributed between aortic dilatation, BAV, PAPVC, CoA, and TGA. A total of 40.7% (11 of 27) of the patients presented with more than 1 cardiac abnormality (Fig. 1 and Table 2).

**Table 2:** Comparison of frequencies of cardiovascular pathologies according to karyotype

	Complete monosomy X	45, X mosaicism	X structural rearrangement	Other, unknown	*P*
N (%)	21 (26)	31 (39)	14 (18)	13 (16)	–
Cardiovascular disorders, N (%)	16 (76)	9 (29)	5 (36)	5 (39)	0.010
CHD, N (%)	15 (71)	8 (26)	5 (36)	5 (39)	0.019
Cardiac surgery, N (%)	5 (24)	2 (7)	1 (8)	1 (8)	0.052
BAV, N (%)	9 (43)	3 (10)	3 (21)	1 (6)	0.01
Aortic coarctation, N (%)	2 (10)	1 (3)	0 (0)	1 (8)	0.181
Aortic dilatation, N (%)	7 (33)	3 (10)	1 (10)	2 (15)	0.039
Aortic regurgitation, N (%)	2 (14)	1 (17)	(0)	1 (13)	0.173
Persistent left superior vena cava, N (%)	2 (10)	1 (3)	0 (0)	0 (0)	0.082
PAPVC, N (%)	6 (29)	0 (0)	0 (0)	0 (0)	<0.001
Arterial hypertension, N (%)	5 (24)	0 (0)	0 (0)	3 (23)	0.137
Hypercholesterolemia, N (%)	1 (5)	0 (0)	0 (0)	1 (8)	0.229

The table shows the repartition of karyotype in the complete cohort of patients with Turner syndrome (N = 79). *P* values are calculated assuming that the 12 patients without imaging had no known congenital heart disease.

BAV, bicuspid aortic valve; CHD, congenital heart disease; PAPVC, partial anomalous venous connection.

#### Monosomy

A total of 51.8% (14 of 27) of the patients with X monosomy had a CHD. Indeed, the most frequent karyotype encountered in TS patient with CHD was pure monosomy of the X chromosome (45, X). Cardiovascular abnormalities were more frequently found in complete monosomy X, with BAV (alone or in association) in 33.3% (9 of 27) patients being the most frequent anomaly, followed by PAPVC in 22.2% (6 of 27) and aortic dilation in 18.5% (5 of 27).

#### Mosaic

Only 22.2% (6 of 27) of the patients with mosaic TS had a CHD.

45 X mosaicism was the most frequent karyotype, representing 39.2% (31 of 79) of the patients of the total cohort of patients with TS. However, it was under-represented in the subgroup of CHD.

#### Structural abnormality

A total of 50% (2 of 4) of the patients with structural abnormality of the chromosome X had a CHD. However, X structural abnormality was relatively rare in our cohort representing only 18% of the patients.

#### Unknown karyotype

A total of 11.1% (3 of 27) of the patients with CHD had an unknown karyotype. Age did not differ between the groups of karyotypes.

### Partial anomalous pulmonary venous connection

PAPVC was found in 9% (6 of 67) of patients and only in the karyotype “complete monosomy X.” In all these patients, echocardiography had missed the PAPVC. PAPVC were diagnosed by CMR when participants reached a median age of 26.6 years (Table 3). A total of 66.7% (4 of 6) of the patients had a right supracardial PAPVC, and 33.3% (2 of 6) had a left supracardial PAPVC (Table 3).

**Table 3:** Patients with PAPVC (N = 6)

Patient number	Type of PAPVC	Qp:Qs	Karyotype	Associated findings	Age at PAPVC diagnosis
1	Right upper lobe vein into the superior vena cava	1.75:1	45 X	–	20 years at second CMR
2	Right upper and segmental tributaries of the middle pulmonary veins into the superior vena cava	1.6:1	45 X	–	33 years at first CMR
3	Right upper lobe vein and segmental tributaries of the middle pulmonary veins into the superior vena cava + left upper lobe vein into the left brachiocephalic vein	1.75:1/0.98∗:1	45 X	–	20 years at first CMR
4	Left upper lobe vein into the left brachiocephalic vein	1.1:1	45 X	–	33 years suspected at echocardiography and confirmed at first CMR
5	Left upper lobe vein into the left brachiocephalic vein	1.6:1	45 X	Dilatation of the right upper pulmonary vein confluent	27 years at first CMR
6	Right upper lobe vein into the superior vena cava	1.3:1	45 X	Persistent left vena cava	27 years at third CMR

CMR, cardiac magnetic resonance; PAPVC, partial anomalous pulmonary venous connection; Qp:Qs, ratio of pulmonary blood flow to systemic blood flow.

∗ Patient number 3 underwent correction of PAPVC. Qp:Qs of 1.75 correspond to the shunt before and 0.98 to the shunt after correction.

However, there were no complications related to PAPVC (no right ventricular [RV] dilatation or valvular disease). Table 4 compares the CMR and echocardiography findings between patients with and without PAPVC.

**Table 4:** Comparison of CMR and echocardiography at last follow-up between patients with and without PAPVC

	No PAPVC (N = 61)	PAPVC (N = 6)	*P*
CMR at last follow-up	33	6	
LV EF (%)	63 ± 5	59 ± 3	0.123
LV end diastolic volume (mL/m^2^)	67 ± 18	62 ± 10	0.432
RV EF (%)	58 ± 8	56 ± 4	0.554
RV end diastolic volume (mL/m^2^)	73 ± 13	81 ± 18	0.291
Qp:Qs	1.03 ± 0.04	1.35 ± 0.24	0.106
Echocardiography	58	6	
LV function (Simpson, %)	66 ± 8	65 ± 6	0.865
RV systolic function			0.905
Normal, N (%)	56 (98)	6 (100)	0.906
Moderate reduced, N (%)	2 (2)	0 (0)	
LV dilatation, N (%)	4 (5)	1 (17)	0.389
RV dilatation, N (%)	0	2 (33)	0.007

Of note, results correspond to last available CMR and TTE. One patient with PAPVC had undergone correction of PAPVC.

CMR, cardiac magnetic resonance; EF, ejection fraction; LV, left ventricle; PAPVC, partial anomalous pulmonary venous connection; Qp:Qs, ratio of pulmonary blood flow to systemic blood flow; RV, right ventricle; TTE, transthoracic echocardiography.

There were no significant differences in terms of biventricular function and ventricular size between the 2 groups (PAPVC vs no PAPVC). In patients with PAPVC, the RV was more frequently dilated (33% vs 0%, *P* = 0.007, OR: 1.5; 95% CI: 0.9; 2.6) on echocardiography, whereas the end-diastolic volume did not differ significantly on CMR (81 ± 18 vs 73 ± 13 mL/m^2^, *P* = 0.291, OR: 0.97; 95% CI: 0.8; 1.2).

The shunt was of minor importance in most patients with PAPVC, with a mean ratio of pulmonary blood flow to systemic blood flow (Qp:Qs) of 1.35 ± 0.24 at last follow-up. However, RV was moderately dilated in only 1 patient known for PAPVC with a significant left-right shunt (Qp:Qs = 1.9). This justified the only PAPVC correction surgery performed in the cohort presented here. The patient was 25 years old at the time of surgery (patient number 3, Table 3).

Another patient also presented a Qp:Qs of 1.75 (patient number 1, Table 3) with a large shunt and RV dilatation. As she also had a BAV with no indication for surgical repair at this time, PAPVC repair was deferred, waiting for BAV surgery.

### Aortic dilatation and BAV

The mean aortic diameter of the total cohort was within the reference range, but aortic dilatation was found in 13 of 39 patients, that is, 32% of those who had undergone CMR. Aortic dilatation was more frequent in patients with BAV (54% vs 17%, *P* = 0.010, OR: 7.5; 95% CI: 2.0; 26.6) and complete monosomy X (63% vs 19%, *P* = 0.013, OR: 4.7; 95% CI: 1.3; 16.3). BAV is known to be significantly associated with increased aortic size.^16^

## Discussion

This study aimed at systematically evaluating the type of cardiac abnormalities found in patients with TS and the cardiovascular outcomes. In this population including children and adults, we found a high proportion CHD, mainly in patients with complete monosomy X.

### General findings

In our center, 83.5% (66 of 79) of patients with TS have undergone an echocardiography at least once in their lifetime and 49% (39 of 79) an additional CMR. Patients with TS appear to be relatively well followed from a cardiological point of view as proposed by established guidelines^17^, ^18^, ^19^ However, it is important to note that PAPVC was missed in all patients by echocardiography and only detected by CMR (Table 3).

### Congenital heart disease

Similar to a study in 2019,^7^ almost half our patients had cardiovascular abnormalities: predominantly CHD lesions with BAV, followed by PAPVC and CoA. Biventricular systolic function was preserved on CMR.

### Partial anomalous pulmonary venous connections

Recently, a higher prevalence of PAPVC than previously thought has been described. Specifically, in a study including 96 patients evaluated with cardiac CT, 25% were found to have PAPVC.^12^ In 2012, Gutmark-Little et al.^20^ conducted a retrospective study to determine the prevalence and hemodynamic significance of PAPVC return in TS using TTE and CMR and found a prevalence of 18% (PAPVC from the right lung in 5 of 7 patients and from the left in 2 of 7 patients). Karyotypes of subjects with PAPVC were complete monosomy X in 57%. Interestingly, and as shown in previous studies, no associated atrial septal defect was present, which is usually typically found in patients with PAPVC without TS.^12^^,^^20^

In another study, Ho et al.^14^ found PAPVC in 13% of the cases in a prospective study of 85 women with TS (self-referred patients). In their study, the PAPVC was most commonly originating from the left upper lobe (8/11) to the left brachiocephalic vein, leading the authors to conclude that this is a distinctive feature of the TS cardiac phenotype because PAPVC usually tends to drain in the coronary sinus or in the inferior vena cava in patients without TS. However, other studies did not confirm this observation. For example, in a retrospective study on 51 patients with TS, Kim et al.^15^ found 15.7% of PAPVC (none diagnosed by previous echocardiography) with 25% having the left upper pulmonary vein drained into the right atrium through a vertical vein and 75% a PAPVC from the right lung.

In our study, PAPVC was encountered with a prevalence of 9% (6 of 67), which is relatively low compared with other studies. This can probably be explained by the fact that only half of our patients underwent CMR. It is also partly explained by the fact that children are less likely to undergo CMR because of the need for sedation. Moreover, in adult TS, echocardiography may be inaccurate for detecting PAPVC because of poor acoustic windows.^21^^,^^22^ The findings in our cohort confirm the limitation of echocardiography, as previously reported,^15^^,^^20^ as we identified PAPVC only by CMR. PAPVC was missed on echocardiography in all our patients, even in those who had had echocardiography during childhood.

The karyotype was complete monosomy X in all the patients with PAPVC, which is concordant with other studies.^12^^,^^14^^,^^23^

The PAPVC were all of the supracardiac type, also in line with the findings of previous studies.^12^^,^^20^ PAPVC originated from the right lung in 67% (4 of 6) of the cases. Interestingly, in our 2 patients with a left PAPVC, the anomalous vein drained in the left brachiocephalic vein, which was in line with the hypothesis of Ho et al.^14^ of a distinctive feature of the PAPVC in patients with TS.

The hemodynamic impact of PAPVC was of minor importance in most of our patients (notably because of the absence of atrial septal defects) as reported by Gutmark-Little et al.,^20^ justifying a conservative approach as proposed by current guidelines.^24^ However, even if only a single patient required an operation for PAPVC, adequate follow-up should be warranted for the early recognition of RV volume overload, RV failure, pulmonary arterial hypertension, and arrythmia.^13^ For these reasons, we adopted the strategy in our institution of performing at least 1 CMR around the age of 18 years, during the transition phase from the pediatric to the adult cardiology unit.

### Valvulopathies and aortic dilatation

We found no significant valvular diseases in our patients except for moderate aortic stenosis in 1 individual. Patients with TS frequently develop dilatation of the aortic root and ascending aorta. The prevalence found in this study, that is, 19.4% (13 of 67) in the whole cohort and 32% of patients who underwent CMR, is similar to that in previous studies.^10^^,^^17^ Of note, we found a clear relationship between the presence of aortic dilatation and arterial hypertension, BAV, and the karyotype. These observations are in line with previous studies describing BAV, the presence of "complete monosomy X,” age, and arterial hypertension as risk markers for progressive aortic dilatation.^10^^,^^17^

### Relationship between karyotype and cardiovascular phenotype

The karyotype in TS women ranges from complete monosomy X to various forms of mosaicism, in which there is a normal cell line or an abnormal second (or third) cell line. As in our study, correlations between genetic patterns and clinical presentations in women with TS have been described previously. For example, females with small distal deletions of the short arm of the X chromosome (Xp22.33) frequently have skeletal anomalies, but they do not appear to be at higher risk for cardiac anomalies. Moreover, 45, X/46, XX mosaicism appears to be associated with a milder cardiovascular phenotype, including less prevalent and less severe CHD.^18^

Regarding the results obtained in this study, the karyotype that seems to be most associated with acquired and congenital cardiovascular abnormalities is complete monosomy X. On the other hand, 45, X, 47, XXX is the karyotype with the least impact on the heart. Women with mosaic TS tend to have fewer signs and health problems than those with classic monosomy. This could be explained by the fact that complete monosomy X results in a more severe loss of chromosomal material than mosaicism, where only a variable subset of DNA is missing in the second X chromosome. On the basis of our findings summarized in Table 2, we suggest that cardiac surveillance is particularly indicated for patients with complete monosomy X.

Echocardiographic follow-up, if feasible under good conditions, is probably sufficient for patients in whom aortic diameters determined by ultrasound correlate well with reference imaging studies such as CMR. However, an initial CMR or CT should be performed in all patients to rule out PAPVC as these imaging modalities are clearly more sensitive for the detection of PAPVC than echocardiography. On the basis of the findings presented here, one could consider increasing the interval between cardiological examinations for mosaic karyotypes, although current surveillance guidelines must still be applied for patients with TS with any karyotype.

Among the 27 patients with CHD, 29% (4 of 14) of patients with monosomy had a history of cardiac surgery. All patients with TS with aortic CoA underwent surgery. These patients were distributed across all karyotype types, with no single karyotype identified as being at greater risk of coarctation.

A more thorough understanding of the molecular mechanism that leads to the phenotypic spectrum according to the various karyotypes might have a significant impact on the screening, the monitoring, and the therapy for associated disorders.^5^

### Limitations

This is a retrospective study, and the data are heterogeneous. In a few patients, the karyotype remains unknown. The sample size is relatively small. Only a limited number of patients had undergone CMR, which is considered the gold standard for evaluation of aortic diameters and pulmonary venous return. This is partly due to the young age of the patients: in children, CT is generally not performed to reduce the exposure to radiation, and CMR is carried out only if there is a suspicion of a cardiac pathology. However, as outlined above, in our institution, we have implemented a policy of performing a CMR in all patients with TS no later than age 18 years (age of transition between pediatric and adult follow-up) to rule out PAPVC and to provide a baseline for comparing vessel diameters later in life, as aortic dilatation can occur even in the absence of BAV or arterial hypertension.^24^

## Conclusions

Almost half of patients with TS present with CHD, and there is a strong association with the underlying karyotype, with a higher prevalence in complete monosomy X compared with patients with mosaicism. Although complete monosomy of the X chromosome is less frequent, these patients need a particularly thorough cardiovascular evaluation and follow-up given that they can present with more serious cardiovascular manifestations that can show progression over time. The most frequent CHD is BAV with or without aortic dilatation, followed by PAPVC and CoA. The impact of PAPVC appears to be minor, rarely requiring surgical repair, unlike CoA. However, echocardiography systematically misses the PAPVC; hence at least 1 CMR examination is recommended in every patient with TS early in life. Ultimately, a better understanding of the genotype-phenotype correlation may allow personalizing the cardiovascular management of patients with TS.
